# Peritoneal Adhesions in Osteopathic Medicine: Theory, Part 1

**DOI:** 10.7759/cureus.42472

**Published:** 2023-07-26

**Authors:** Bruno Bordoni, Allan R Escher, Gregory T Girgenti

**Affiliations:** 1 Physical Medicine and Rehabilitation, Don Carlo Gnocchi Foundation, Milan, ITA; 2 Anesthesiology/Pain Medicine, H. Lee Moffitt Cancer Center and Research Institute, Tampa, USA; 3 Anesthesiology, H. Lee Moffitt Cancer Center and Research Institute, Tampa, USA

**Keywords:** peritoneum, adherence, scar, pain, myofascial, osteopathy, osteopathic manipulation, fascia

## Abstract

Peritoneal adhesions form as a result of trauma to the abdomen, injuries resulting from surgery, and infections. These tissutal neoformations are innervated and vascularized, and with lymphatic vessels, adherence becomes a new and independent structure, capable of negatively influencing visceral functions. Adherent neogenesis can be asymptomatic or can be a source of pain, limiting the patient's quality of life. Although adhesiolysis remains the elective approach to eliminate adhesions, this therapeutic route prepares the peritoneal anatomical area to recur. The article reviews information on adhesion formation and peritoneal anatomy, probable subjective predispositions, and pathways that carry nociception. The text aims to be a theoretical basis for making new treatment suggestions for non-invasive osteopathic medicine, through a second part will be discussed in another article.

## Introduction and background

The adherence between different tissues is a physiological process of repair after a trauma, a surgical approach, or an infection. This tissutal response is hyperplasia that can involve any area of the body (from the dermis down), between the viscera, between the serous cavities and the viscera, between tendons and muscles, between soft tissues and joints, between the different layers of the nerve, and between the vascular pathways and the dural layer [1,2]. If the adherence becomes a source of pain and/or causes an alteration of the mechano-metabolic function, the hyperplasia becomes a pathological adherence [1,2]. The finding of such formations is variable. In America, 20 million patients undergoing invasive surgery in one year have a 95% risk of producing adhesions [1]. Compared to the surgical site, peritoneal adhesions can form at a percentage of 90%, which can cause chronic pain, female infertility, and intestinal obstruction [1]. Adhesiolysis is often the treatment of choice, but with such re-surgery, a new adhesion formation is a repeating event (approximately 80%) [1]. The presence of these formations extends the surgical times, increasing the peri- and post-surgical dangers for the patient [1]. Adhesions can be a source of unexplained pain after surgery not only at the peritoneal level, such as post-sternotomy pain syndrome, which is found in up to 66% of patients undergoing thoracic cardiac surgery, with possible consequences in the increased risk of injury to large vessels, heart, lung, detection of Ileus, and heart failure [1,3]. Adhesions may be one of the causes of failed back surgery syndrome, which affects approximately 76% of patients undergoing spinal surgery, peripheral nerve conduction abnormalities, and dural lesions [1,4]. The presence of adhesions to the uterus following invasive surgery or the application of intrauterine devices, found in over 60%, can cause alterations in the menstrual cycle, placental dysfunction, Asherman's syndrome (obliterated uterus), infertility, and psychological problems [1]. The tissues involved in the repair/inflammation process are unable to slide with each other; adhesion interrupts the physiological sliding movement [2]. This area of fixity generates new vectors of movement, which alter the perception of the central and peripheral nervous systems of the same areas [5]. The nervous system will receive non-physiological proprioceptive afferents (interoception and exteroception). This chronically altered bodily perception will negatively affect other systems, such as the motor, emotional, and nociceptive systems [6,7]. The mechanotransductive processes of the tissues involved in adherence will be altered, making the ability of cells to respond adequately to the mechanical stimuli they undergo less efficient [5]. The latter concept translates as a weaker tissue (less ability to adapt), despite apparently the tissue having greater stiffness. The adhesions formed are innervated and vascularized with lymphatic vessels [8]. These events occur within a week of injury, making the different tissue layers not only contiguous but also continuous [8]. This is one of the most important reasons why the separation of the layers in the presence of adhesions can only be performed surgically and not with manual medicine. The article presents the most current information on the mechanisms that allow the formation of post-surgical or traumatic adhesions related to infections/inflammations, about the peritoneum, the largest serous membrane of the human body. The text briefly highlights any precautions of a preventive nature and hints at the peritoneal anatomy, as well as possible individual predispositions or risk factors that could create a favorable environment for adhesion neogenesis. Finally, the article describes the preferential pathways that carry nociception from adhesions to areas of the central nervous system. In the next article (second part), we will discuss the instrumental and manual evaluation for the detection of adhesions and the possible symptomatological pictures regardless of the presence of pain in the surgical or injured area; finally, we will propose possible treatments in the field of osteopathic medicine. The latter approach aims not to break the adhesions but to improve the possible symptomatic pictures that can derive from a peritoneal adhesion, such as pain or visceral dysfunction.

## Review

Prevention

The concept on which post-surgical prevention is based is to prevent the layers of tissue subjected to surgical injury from remaining adherent and immobile. There are different barrier systems and drugs to avoid the formation of adhesions. Generally, the barriers placed by the surgeon between the layers involved in the invasive operation are made up of biomaterials that are reabsorbed over time [1,9,10]. The goal is to prevent tissue neogenesis. These barriers or films (electrospun fibrous mats, nanospheres, and hydrogels of various natures) may contain drugs, with the further intention of slowing down the inflammation processes and inhibiting the reaction of the fibroblasts to the surgical insult [1,9-11]. The focus of the article goes beyond the analysis of different therapeutic means used for prevention, and for further analysis of such pharmacological approaches and barriers, readers can read the work of Kheilnezhad and Hadjizadeh and Liao [1,12]. Tissue immobilization after surgery is essential to ensure tissue integrity. Unfortunately, a precaution such as the immobility of the surgical area is one of the causes that irreversibly create adherence, which forms after three to five days [1,13]. The surgery itself slows peristalsis (less movement), as do opioids [14]. The lack of tissue sliding generates inflammation and opens the door for tissue neogenesis [15,16]. The ideal would be to be able to apply movements to maintain the sliding of the tissues during the patient's stay. Animal studies demonstrate that manual manipulations on the first day after surgery and on the abdominal area are able to reduce (but not eliminate) the formation of peritoneal adhesions [14,17]. We do not know what would happen in the human model, and we have no follow-up data from the animal model or if the patient can tolerate manipulations on the abdominal area the day after surgery. Another preventive approach is inherent in the surgical act, that is, using the utmost delicacy (when possible) or the least invasiveness possible. But still, we do not have enough concrete data to state that mini-surgery and robotic use for surgery are able to minimize adhesion formation [18]. Gene therapy is another developing area, with the aim of adequately inhibiting certain genes to reduce the excessive inflammatory response, but we do not yet have enough data to make any claims [18].

Peritoneum

The peritoneum derives from the mesoderm, from which we will find the mesothelium (the layer that covers the coelom) and the mesenchyme; development starts in the phase of gastrulation from the primitive intestine [19]. The layer that continues to cover the primitive intestine will form the visceral peritoneum, while the mesenchyme that covers the external area will develop into the parietal peritoneum [19]. The process of closing the primitive intestine will unite the two layers (inner and outer) to form the mesentery. The latter will form the different visceral ligaments of the abdomen, connecting different viscera: splenorenal ligament, gastro-splenic ligament, liver ligaments, and large and small caul [19]. The caudal peritoneal area will form the apex of the pelvic cavity and, in women, will develop a space between the rectum and uterus (Douglas pouch) [19]. A space will remain between the parietal and visceral layer, in which a fluid of about 5-100 milliliters will circulate inside, deriving from the ultrafiltration of the blood; the drainage of this fluid will be by numerous lymphatic stomata [19]. The peritoneum is the largest of the serous cavities of the human body [20]. The visceral peritoneum covers the external surface of the viscera and the mesenteric surface of the abdomen, while the parietal peritoneum covers the internal area of the abdomen, anteriorly and posteriorly, and the internal pelvic surface, the two layers form the peritoneal cavity [20]. The parietal peritoneum is covered by a film of mesothelial cells (MCs) (reminiscent of epithelial cells), and underlying the latter, we find a basement membrane and a sub-peritoneal stroma (containing fibroblasts, adipocytes) [21]. The presence of MCs allows them to obtain a smooth and non-adhesive surface, preventing any friction between the two layers; these cells form a first barrier against toxic substances, and being this semi-permeable barrier, it allows the passive passage of multiple ionic substances [21,22]. MCs have intercellular junctions (zonula occludens and zonula adherens, desmosomes, gap junction), apical-basal polarity, and microvilli. The junctions allow to keep the peritoneal structure stable and protected, and the polarity allows to maintain the shape and function of the peritoneum [19,23,24]. Microvilli create a lubricant for tissue gliding and contribute to barrier function [19,20]. The innervation system is very complex. The efferents involve the sympathetic and vagal systems, while the non-separable afferents between the adrenergic and cholinergic systems project toward lamina I and lamina V dorsally [25]. The parietal peritoneum contains receptors capable of sending signals related to pain, pressure, touch, temperature, and injuries that negatively affect structural integrity [25]. The anterolateral area receives sensory branches from intercostal nerves (6 to 8), while the central parietal peritoneal area (below the diaphragm) receives sensory afferents from the phrenic nerve, vagus nerve, and branches of the sympathetic system [25]. The remaining area is innervated by sympathetic afferents [25]. The pelvic area of the parietal peritoneum receives branches from the lumbar plexus (L2-L4) from the obturator nerve [25]. The innervation that reaches the parietal peritoneum reflects the dermatomal organization, starting from T6 up to T12 and L2-L4. T6 involves the skin of the xiphoid, and area T10 corresponds to the periumbilical area; finally, area T12 involves the abdomen area inferiorly to the pubic area [25]. Some parietal peritoneal afferents contribute to proprioceptive information (visceroception-interoception), probably by contraction and distension movement of the abdomen [25]. The innervation of the sensory visceral peritoneum is by the spinal nerves and the vagus nerve; the stresses that stimulate the afferents from the visceral peritoneum are of the mechanical type, such as stretching and deformation [25]. The mesentery (fan-shaped fold of peritoneum) is affected by spinal nerves from T5 to T12; the anatomical area involves the duodenojejunal flexure, the oesophagogastric junction up to the mesorectum [25,26].

Pathogenesis of adhesions

**Figure 1:**
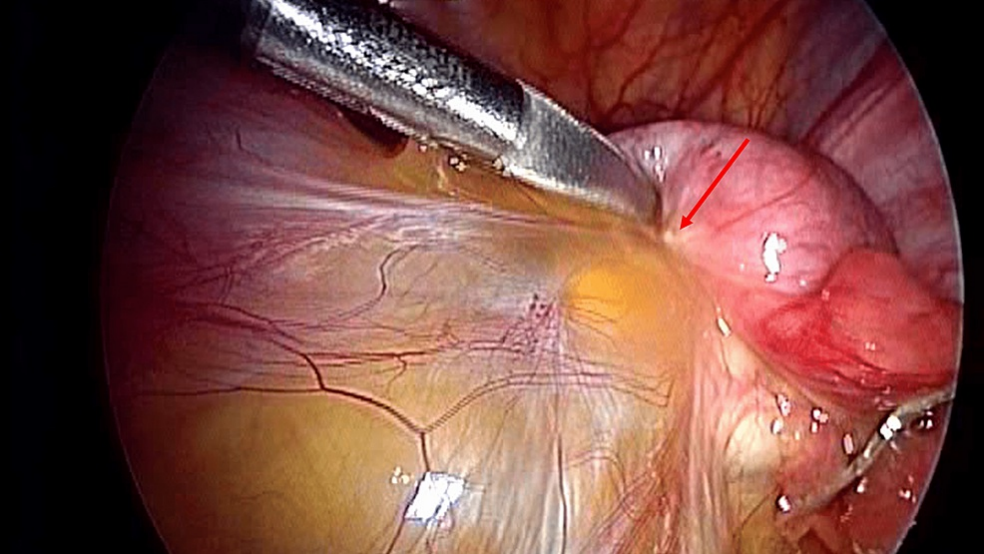
The image shows an adhesion formation due to a previous inflammation between the sigmoid and the surrounding tissue The image is owned by Bordoni Bruno.

Adhesions are scar tissue with inhomogeneous morphology and thickness [27]. They can appear as thin or thick and translucent films or strings with variable lengths; they are innervated and vascularized, containing adipose tissue, collagen, and inflammatory cells [27]. The adhesions can be asymptomatic for the patient and sometimes come to light only with surgery; moreover, an inflammatory state without a previous surgical lesion can be the cause of adhesion formation, such as for previous peritonitis or endometriosis, the use of radiotherapy, peritoneal dialysis (Figure 1) [27].

When the integrity of the peritoneum is compromised by injury or by the constant presence of inflammation or infection, the involved peritoneal surface can undergo exposure to the surrounding tissues. On the one hand, the lubricating structures are altered (proteoglycans, glycosaminoglycans, phospholipoprotein surfactant complexes) causing future frictions [22,27]. On the other hand, trauma stimulates the peritoneum to release damage-associated molecular patterns (DAMPs) or pathogen-associated molecular patterns (PAMPs) to recruit immune system cells such as neutrophils and macrophages [20,27]. The recruitment of immune cells occurs within hours of the traumatic event. The intact peritoneum adjacent to the denuded one is negatively affected due to the friction present; this event leads to the formation of adherent tissue involving areas of healthy tissue [13]. Within hours of the traumatic event, there is an imbalance of genesis between plasminogen activator inhibitor-1 (PAI-1) and tissue plasminogen activator (tPA); the former increases, while the latter decreases. This poorly understood mechanism leads to the accumulation of fibrin and adherence [28]. Numerous biochemicals are involved, such as substance P (acting via the neurokinin type 1 receptor); substance P stimulates the activation of interferon-gamma (secreted in turn by natural killer cells), which later promotes the synthesis of PAI-1 [28]. Recalled neutrophils (predominant leukocytes) can be activated by both interferon-gamma and substance P [28]. Other substances will affect the formation of adhesions, such as the increase of vascular endothelial growth factor and interleukins (type 6 and 17) [27]. DAMPs and PAMPs stimulate inflammation, and the first reaction is coagulation and increased permeability of vessels [20]. The fibrin released by the more permeable vessels will be a support structure for the fibroblasts, which will synthesize collagen and adhesions [20]. Peritoneal MCs will detach from the basement membrane and enter the peritoneal cavity; this occurrence will stimulate the synthesis of extracellular matrix (invading the basement membrane) [20]. MCs will bring other inflammatory cells, such as macrophages, and will stimulate the activation of substances that decrease the ability to repair (transforming growth factor 1-β, tumor necrosis factor-alpha) and perpetuate the inflammatory status [20]. We also know that MCs will undergo a regression, transforming into mesenchyme and then into myofibroblasts [20,27]. The latter mechanism occurs by several actors, such as transforming growth factor 1-β, interleukin type 1, angiotensin, hypoxia-inducible factor 1-alpha, and heparin-binding growth factor type 8 (pleiotrophin) [27]. Myofibroblasts will mainly express collagen types I and III [29]. Mesenchymal cells in an inflammatory environment will recall substances capable of stimulating adhesion, such as intercellular adhesion molecule-1 and vascular cell adhesion molecule-1 [20]. Currently, we do not know in detail the biochemical pathways that favor the formation of permanent adhesions [29].

​Classification of adhesions

Based on the period that elapses after the peritoneal traumatic event, some authors try to give a classification of the adhesions or phases in which they form. Membranous adhesions are those formed by a large amount of fibrin deposits, clots, and a strong inflammatory environment that persists; this typology is found in the first three days [1]. Vascular adhesions (days 3 to 21) are composed of loose connective tissue, MCs, fibroblasts and myofibroblasts, collagen, and blood/lymphatic vessels [1]. Adhesive adherence is the third formation that is created approximately in the fourth week after the injurious event. It consists mainly of type I collagen, myofibroblasts, endothelial cells, and nerve branches [1]. The last phase of adhesion formation, which action can continue for months and years, is referred to as scar adhesion, with blood vessels (arterioles, venules, and capillaries), lymphatic vessels, and nerves fully formed and a constant presence of inflammatory cells [1]. Adherence is, therefore, a non-transient formation and in a state of chronic inflammation. The presence of a hematoma could be predictive of adhesion formation [1]. The increased stiffness that derives from this formation causes a self-feeding of the adherence; moreover, the adherence is sensitive to mechanical variations [1]. This increased mechanical sensitivity (from stretching to vigorous massage) stimulates the fibroblasts to constantly synthesize myofibroblasts, resulting in adherence and an inflammatory environment [1].

Predisposition

In the literature, we find some clinical conditions that could favor the emergence of adhesions: patients with a situation of chronic inflammatory status, genetic factors, use of antibiotics, post-surgery blood transfusions, obesity, smoking and excessive alcohol intake, presence of infections, metabolic pathologies, the menstrual cycle (in the post-ovulatory phase), the presence of sexually transmitted diseases, high cholesterol values, emotional stress, and pregnancy. These conditions can alter one or more factors involving the immune response, blood coagulation, and tissue fibrinolytic capacity [22,30-32].

Paths of pain

As described earlier in the article, the peritoneum is richly innervated. The adhesions that form are also innervated. In animals, it has been found that innervation can derive not only from the peritoneum but also from surgically injured visceral tissue and from the abdomen. Complex ramifications can arise from the enteric system and the muscular system of the abdomen, crossing the adherence [33]. These fibers carry nociceptive information [33]. Somatic fibers involve the skin, muscles, and parietal peritoneum [33]. Probably, it is the blood vessels that guide the axonal growth/direction; first, vascular neogenesis would occur and then axonal neogenesis [33]. These fibers capable of transmitting pain (myelinated and unmyelinated) are found in patients with peritoneal adhesions, regardless of the presence of pain felt by the patient and regardless of the surgical area and the amount of these branches [34]. These axonal branches are connected to mechanical receptors with different activation thresholds; when the receptors are activated by mechanical stimuli (distension, stretching, etc.), on the one hand, they can stimulate further inflammation due to a probable antidromic release of neuropeptides, and on the other hand, they can send nociceptive information [34]. Nociceptive information will pass through different areas of the spinal cord (dorsal and ventral laminae) and will be transported to higher centers bilaterally, i.e., nociception originating from a right abdominal area will also be processed by the contralateral medullary laminae, and vice versa [35]. This concept is based on the theory of distributed pain (bilateral pathways), with respect to the view of a serial transmission [35]. This vision complicates the understanding of the origin of the symptom, and, moreover, the nervous system could send altered responses such that the patient is unable to find psychophysical well-being [36]. The preferential route of transmission will be the spinothalamic route, but other brain areas will receive the same information, such as the cerebellar and vestibular areas, the limbic area, the primary and secondary motor and sensory cortex, and the anterior cingulate cortex [35]. We do not know in detail how nociceptive information is processed, not only due to lack of data but also because each patient has a subjective clinical path.

## Conclusions

In this first part, the article has presented the most current information on the mechanisms that allow the formation of post-surgical or traumatic adhesions, or those linked to infections/inflammations, with regard to the peritoneum, the largest serous membrane of the human body. We have reported information on possible precautions of a preventive type and references on peritoneal anatomy, as well as possible individual predispositions or risk factors that could create a favorable environment for adhesion neogenesis. Finally, the text discussed the preferential pathways that carry nociception from adhesions to areas of the central nervous system. We can deduce from the above that adhesions live in an inflammatory environment and arise from a complex process of inflammation. The presence of inflammation suggests that the osteopathic manual approach should be non-invasive but as gentle as possible so as not to foment further immune responses.
